# Serum sodium level is inversely associated with new‐onset diabetes in hypertensive patients

**DOI:** 10.1111/1753-0407.13338

**Published:** 2022-12-05

**Authors:** Qi Cheng, Xiaocong Liu, Anping Cai, Dan Zhou, Yuqing Huang, Yingqing Feng

**Affiliations:** ^1^ Department of Cardiology Guangdong Cardiovascular Institute, Guangdong Provincial People's Hospital, Guangdong Academy of Medical Sciences Guangzhou Guangdong China; ^2^ Hypertension Research Laboratory, Guangdong Provincial Clinical Research Center for Cardiovascular Disease Guangdong Cardiovascular Institute, Guangdong Provincial People's Hospital, Guangdong Academy of Medical Sciences Guangzhou Guangdong China

**Keywords:** hypertension, new‐onset diabetes, serum sodium, 血清钠, 新发糖尿病, 高血压

## Abstract

**Background:**

Serum sodium level is associated with cardiovascular and endocrine health. Though decreased serum sodium is considered to be associated with reduced hypertension risk, some studies also found that it may increase the risk of diabetes. This study aimed to investigate the association of serum sodium with new‐onset diabetes in hypertensive patients.

**Methods:**

Based on the annual health examinations from 2011 to 2016 in Dongguan City, Guangdong, China, hypertensive patients without diabetes at baseline were selected. Logistic regression and restricted cubic spline were used to evaluate the association between serum sodium level and new‐onset diabetes. Subgroup analysis was also conducted.

**Results:**

A total of 4438 hypertensive patients with a mean age of 58.65 years were included, of whom 48.9% were male. During a median follow‐up of 35.1 months, 617 (13.9%) of the subjects developed new‐onset diabetes. Per 1‐SD (3.39 mmol/L) increment of serum sodium was associated with a 14% lower risk of new‐onset diabetes (odds ratio = 0.86; 95% CI: 0.78, 0.97; *p* = 0.01). The lowest quartile of serum sodium was associated with the lowest diabetes risk. The restricted cubic spline showed a linear inverse relationship (nonlinear *p* = 0.72). Across all the subgroups, the inverse association was consistent (*p* for interaction >0.05).

**Conclusion:**

An inverse association of serum sodium with new‐onset diabetes in hypertensive patients was observed.

## Graphical abstract

**Highlights**

- There is an inverse association between serum sodium and new‐onset diabetes in a Chinese community‐based hypertensive population.
- Sodium control may need to be further refined to provide comprehensive cardiovascular and diabetes risk management for hypertensive patients.

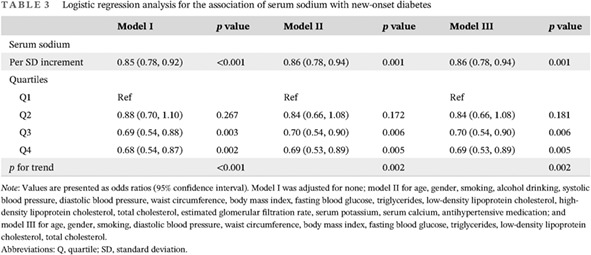

## 摘要

背景:血清钠水平与心血管和内分泌健康相关。虽然降低血钠被认为与降低高血压风险相关, 但也有研究发现其可能增加糖尿病风险。本研究旨在探究血清钠与高血压患者新发糖尿病的关系。

方法:基于2011‐2016年广东省东莞市年度健康体检人群, 选取基线时无糖尿病的高血压患者。采用Logistic回归和限制性立方样条分析血钠水平与新发糖尿病的关系, 并进行亚组分析。

结果:研究共纳入4438例高血压患者, 平均年龄58.65岁, 男性占48.9%。中位随访35.1个月, 617例(13.9%)研究对象新发糖尿病。血钠每增加1SD (3.39 mmol/L), 新发糖尿病风险降低14%。(or = 0.86;95% ci: 0.78, 0.97;p值= 0.01)。血清钠的最低四分位数与最低的糖尿病风险相关。限制性立方样条呈线性反比关系(非线性p值= 0.72)。在所有亚组中, 负相关是一致的(交互作用的p值>0.05)。

结论:血清钠与高血压患者新发糖尿病呈负相关。

## 1 INTRODUCTION

Hypertension and diabetes are two of the most common chronic diseases worldwide. According to epidemiological reports, the global prevalence rate of hypertension was estimated to be 29% in 2025 and 4.4% in 2030 for diabetes, presenting an increasing trend.^1^, ^2^ Compared to the healthy population without hypertension and diabetes or patients with hypertension alone, hypertension combined with diabetes can multiply cardiovascular risk.^3^ Common risk factors like aging, overweight, smoking, and dyslipidemia can induce the incidence of both hypertension and diabetes. Since the hypertensive population is large, managing risk factors and preventing hypertensive patients from developing diabetes is essential.

Serum electrolytes play important roles in physiological mechanisms like balancing osmotic pressure, regulating muscle contraction, and nerve transmission. They also affect blood pressure and glucose metabolism. For example, serum potassium is inversely associated with new‐onset diabetes in hypertensive patients independent of diuretic use.^4^, ^5^ Lower serum magnesium induces insulin resistance and activates thromboxane synthesis, which increases both diabetes and hypertension risk.^6^, ^7^ Increased serum calcium can increase diabetes risk by interfering with the calcium‐dependent process of insulin release.^8^

Sodium is also an important factor in the circulatory and endocrine system, but studies exploring the association between serum sodium and new‐onset diabetes are lacking, especially in the hypertensive population. It is universally acknowledged that reducing dietary sodium intake prevents hypertension. The recommended sodium intake is lower than 2400 mg/day in China.^9^ Dietary sodium is associated with serum sodium,^10^ and serum sodium is also known as a factor that could increase the risk of hypertension.^11^ However, some studies found that lower serum sodium, even within the normal range, is associated with poor prognosis.^12^, ^13^, ^14^ Meanwhile, hyponatremia is independently associated with an increased risk of diabetes.^15^ Sodium and glucose are the main contributors that activate neurohumoral regulation, maintaining a stable range of osmolality.^16^ Thus, we hypothesized that serum sodium levels could affect glucose metabolism, which in turn might reflect diabetes risk. This study aimed to explore the association between serum sodium and new‐onset diabetes in a Chinese community‐based hypertensive population.

## 2 METHODS

### 2.1 Study population

**FIGURE 1:**
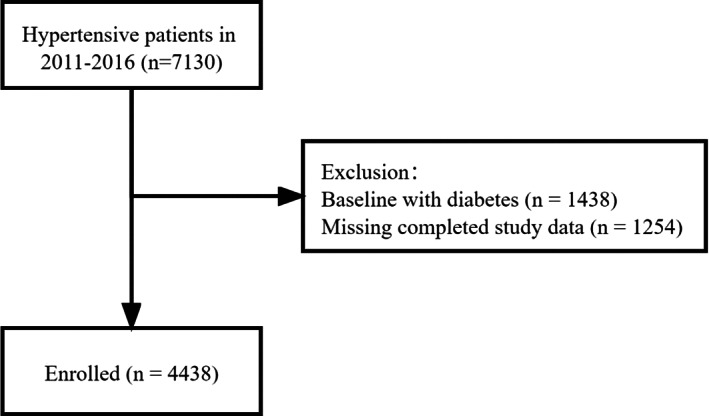
The study flow chart

The study population was Asian adults diagnosed with essential hypertension from Liaobu County of Dongguan City, Guangdong, China. Overall, 7130 patients who participated in annual health examinations between 2011 and 2016 at least twice with examination intervals of more than 1 year were included. After excluding patients with diabetes and subjects without completed laboratory results, a total of 4438 individuals were enrolled in the formal analysis (Figure 1). Guangdong Provincial People's Hospital's medical ethical committee approved the investigation (20110104H). All participants signed informed consent.

### 2.2 Data collection and definitions

We applied standard questionnaires to collect demographic information including date of birth, gender, the status of smoking and drinking, medical history, and current medication. For physical examination, height, weight, waist circumference, systolic blood pressure (SBP) and diastolic blood pressure (DBP) were examined. Body mass index (BMI) was calculated as weight (kg) divided by height squared (m^2^). By processing fasting blood samples in the central laboratory, the concentration of fasting blood glucose (FBG), low‐density lipoprotein cholesterol (LDL‐C), high‐density lipoprotein cholesterol (HDL‐C), total cholesterol (TC), triglycerides (TG), serum creatinine, serum sodium, serum potassium, and serum calcium were tested with a biochemical analyzer (Hitachi 7170A autoanalyzer, Tokyo, Japan). The calculation of the estimated glomerular filtration rate (eGFR) was based on age, sex, ethnicity, and serum creatinine, using the Chronic Kidney Disease Epidemiology Collaboration (CKD‐EPI) creatinine equation.^17^ Diabetes history was defined as having been diagnosed with diabetes previously, receiving hypoglycemic therapy, or FBG ≥7.0 mmol/L at baseline visit.^18^ The diagnostic criteria for hypertension included measuring SBP ≥ 140 mm Hg and/or DBP ≥ 90 mm Hg three times on different days, self‐reported hypertension history, or taking antihypertensive drugs.^19^

The exposure variable was serum sodium at baseline. As previous studies observed, serum sodium has shown heritability and individuality, which may explain the reason why the serum sodium levels of a particular individual can remain in a narrow range in the long term.^20^ Thus, we assumed that the serum sodium level would be stable with only minor dynamic changes, and it could predict health status in reasonable follow‐up time.

### 2.3 Outcome

The outcome was new‐onset diabetes. Subjects without diabetes history at baseline were identified as having developed new‐onset diabetes if FBG tested ≥7.0 mmol/L at subsequent follow‐up visits, if they were diagnosed with new‐onset diabetes by medical institutions, or if they started hypoglycemic therapy during the follow‐up period.

### 2.4 Statistical analysis

During follow‐up, the participants who developed new‐onset diabetes were classified as the new‐onset diabetes group, the others as the non‐diabetes group. Baseline characteristics between the two groups were compared. The levels of serum sodium were divided into quartiles (Q1, Q2, Q3, Q4). Baseline characteristics and incidence of diabetes were also compared across the groups classified by serum sodium quartiles. For continuous variables, data were presented by mean value ± standard deviation and compared by Student's *t* test or Wilcoxon rank sum test. Categorical variables were presented as frequency (percent) and compared by chi‐square tests. To evaluate the associations between serum sodium quartiles and new‐onset diabetes, logistic regression in three models was conducted. Model I was not adjusted for any variables. Model II incorporated variables including age, gender, smoking and drinking status, SBP and DBP, waist circumference, BMI, FBG, TG, LDL‐C, HDL‐C, TC, eGFR, serum potassium, serum calcium, and antihypertensive medication. Using both forward and backward stepwise regression, the significant covariates from model II were selected for adjustment in model III. Restricted cubic spline was applied to detect the nonlinear relationship between serum sodium and new‐onset diabetes. Subgroup analyses for age, sex, BMI, baseline FBG, and antihypertensive drug usage were also conducted. All statistical analyses were performed using R software (version 4.1.2; R Foundation for Statistical Computing, Vienna, Austria). Statistical significance was determined by a two‐sided *p* < 0.05.

## 3 RESULTS

### 3.1 Baseline characteristics

The baseline characteristics according to diabetes outcome and quartiles of serum sodium are presented in Tables 1 and 2, respectively. Of all selected individuals, 48.9% were males, and the mean age was 58.65 ± 13.84 years. The average SBP was 138.65 ± 16.84 mm Hg, and DBP was 85.23 ± 10.86 mm Hg. Overall, 71.5% of participants were on antihypertensive medication. During the median follow‐up of 35.1 months, 617 (13.90%) of the hypertensive subjects developed new‐onset diabetes. Compared with the non‐diabetes group, the patients who developed diabetes were older, more likely to be females, had higher SBP, waist circumference, and BMI. The new‐onset diabetes group had higher FBG, TG, and LDL‐C but lower serum sodium. The use of different types of antihypertensive drugs did not differ significantly between the non‐diabetes group and the new‐onset diabetes group (Supplementary Table S1). The difference in baseline characteristics among quartiles of serum sodium was not significant, except for age and eGFR.

**TABLE 1:** Comparison of baseline characteristics between non‐diabetes group and new‐onset diabetes group

	Total	Non‐diabetes group	New‐onset diabetes group	*p* value
*n*	4438	3821	617	
Age, years	58.65 ± 13.84	58.40 ± 13.83	60.22 ± 13.80	0.002
Age groups, *n* (%)				0.001
<40 years	343 (7.7)	303 (7.9)	40 (6.5)	
40 ~ 50 years	1077 (24.3)	958 (25.1)	119 (19.3)	
50 ~ 60 years	1003 (22.6)	842 (22.0)	161 (26.1)	
60 ~ 70 years	963 (21.7)	841 (22.0)	122 (19.8)	
70 ~ 80 years	730 (16.4)	612 (16.0)	118 (19.1)	
> = 80 years	322 (7.3)	265 (6.9)	57 (9.2)	
Gender (male), *n* (%)	2170 (48.9)	1893 (49.5)	277 (44.9)	0.032
Smoking, *n* (%)	1047 (23.6)	897 (23.5)	150 (24.3)	0.65
Alcohol drinking, *n* (%)	543 (12.2)	469 (12.3)	74 (12.0)	0.843
SBP, mm Hg	138.65 ± 16.84	138.41 ± 16.73	140.14 ± 17.48	0.018
DBP, mm Hg	85.23 ± 10.86	85.27 ± 10.71	84.98 ± 11.76	0.548
WC, cm	87.36 ± 9.13	86.82 ± 8.96	90.74 ± 9.40	<0.001
BMI, kg/m^2^	25.15 ± 3.61	24.95 ± 3.51	26.43 ± 3.96	<0.001
FBG, mmol/L	4.90 ± 0.65	4.82 ± 0.60	5.38 ± 0.73	<0.001
TG, mmol/L	1.94 ± 1.63	1.86 ± 1.48	2.41 ± 2.30	<0.001
LDL‐C, mmol/L	2.78 ± 0.80	2.76 ± 0.79	2.89 ± 0.84	<0.001
HDL‐C, mmol/L	1.31 ± 0.36	1.31 ± 0.35	1.31 ± 0.43	0.991
TC, mmol/L	5.15 ± 1.11	5.13 ± 1.11	5.22 ± 1.15	0.075
eGFR, ml/min	86.97 ± 22.00	87.14 ± 22.04	85.89 ± 21.72	0.189
Serum sodium, mmol/L	141.07 ± 3.39	141.14 ± 3.38	140.59 ± 3.36	<0.001
Serum potassium, mmol/L	4.07 ± 0.43	4.07 ± 0.43	4.09 ± 0.41	0.318
Serum calcium, mmol/L	2.33 ± 2.69	2.34 ± 2.90	2.30 ± 0.26	0.748
Antihypertensive medication, *n* (%)	3173 (71.5)	2732 (71.5)	441 (71.5)	0.99

*Note*: Values are presented as mean ± standardized differences or *n* (%).

Abbreviations: BMI, body mass index; DBP, diastolic blood pressure; eGFR, estimated glomerular filtration rate; FBG, fasting blood glucose; HDL‐C, high‐density lipoprotein cholesterol; LDL‐C, low‐density lipoprotein cholesterol; *n*, number; SBP, systolic blood pressure; TC, total cholesterol; TG, triglycerides; WC, waist circumference.

**TABLE 2:** Baseline characteristics of study participants according to quartiles of serum sodium

Serum sodium, mmol/L	Q1: 122.6–138.4	Q2: 138.5–140.8	Q3: 140.9–143.5	Q4: 143.6–166.7	*p* value
*n*	1082	1120	1112	1124	
Age, years	59.75 ± 14.00	58.36 ± 13.55	57.96 ± 13.95	58.56 ± 13.82	0.017
Gender (male), *n* (%)	529 (48.9)	541 (48.3)	574 (51.6)	526 (46.8)	0.142
Smoking, *n* (%)	267 (24.7)	265 (23.7)	270 (24.3)	245 (21.8)	0.39
Alcohol drinking, *n* (%)	130 (12.0)	141 (12.6)	134 (12.1)	138 (12.3)	0.975
SBP, mm Hg	139.30 ± 16.87	138.51 ± 17.11	138.71 ± 17.28	138.10 ± 16.10	0.406
DBP, mm Hg	84.95 ± 10.64	85.08 ± 10.89	85.84 ± 10.83	85.03 ± 11.08	0.185
WC, cm	87.45 ± 9.51	87.45 ± 9.40	87.16 ± 8.70	87.41 ± 8.88	0.857
BMI, kg/m^2^	25.29 ± 3.71	25.24 ± 3.66	24.96 ± 3.44	25.12 ± 3.64	0.145
FBG, mmol/L	4.87 ± 0.64	4.91 ± 0.66	4.91 ± 0.63	4.90 ± 0.65	0.486
TG, mmol/L	2.00 ± 1.62	1.96 ± 1.76	1.88 ± 1.45	1.92 ± 1.66	0.284
LDL‐C, mmol/L	2.79 ± 0.80	2.81 ± 0.83	2.77 ± 0.82	2.72 ± 0.74	0.054
HDL‐C, mmol/L	1.31 ± 0.37	1.31 ± 0.35	1.31 ± 0.38	1.31 ± 0.36	0.961
TC, mmol/L	5.07 ± 1.07	5.18 ± 1.15	5.15 ± 1.15	5.17 ± 1.07	0.102
eGFR, ml/min	85.42 ± 22.23	87.34 ± 22.02	86.97 ± 21.01	88.09 ± 22.63	0.035
Serum sodium, mmol/L	136.77 ± 1.17	139.64 ± 0.68	142.21 ± 0.79	145.49 ± 1.49	<0.001
Serum potassium, mmol/L	4.06 ± 0.41	4.08 ± 0.43	4.09 ± 0.43	4.07 ± 0.43	0.323
Serum calcium, mmol/L	2.40 ± 4.14	2.28 ± 0.26	2.28 ± 0.17	2.38 ± 3.46	0.594
Antihypertensive medication, *n* (%)	753 (69.6)	792 (70.7)	817 (73.5)	811 (72.2)	0.201
New‐onset diabetes, *n* (%)	180 (16.6)	167 (14.9)	135 (12.1)	135 (12.0)	0.003

*Note*: Values are presented as mean ± standardized differences or *n* (%).

Abbreviations: BMI, body mass index; DBP, diastolic blood pressure; eGFR, estimated glomerular filtration rate; FBG, fasting blood glucose; HDL‐C, high‐density lipoprotein cholesterol; LDL‐C, low‐density lipoprotein cholesterol; *n*, number; Q, quartile; SBP, systolic blood pressure; TC, total cholesterol; TG, triglycerides; WC, waist circumference.

### 3.2 Association of serum sodium with new‐onset diabetes

**FIGURE 2:**
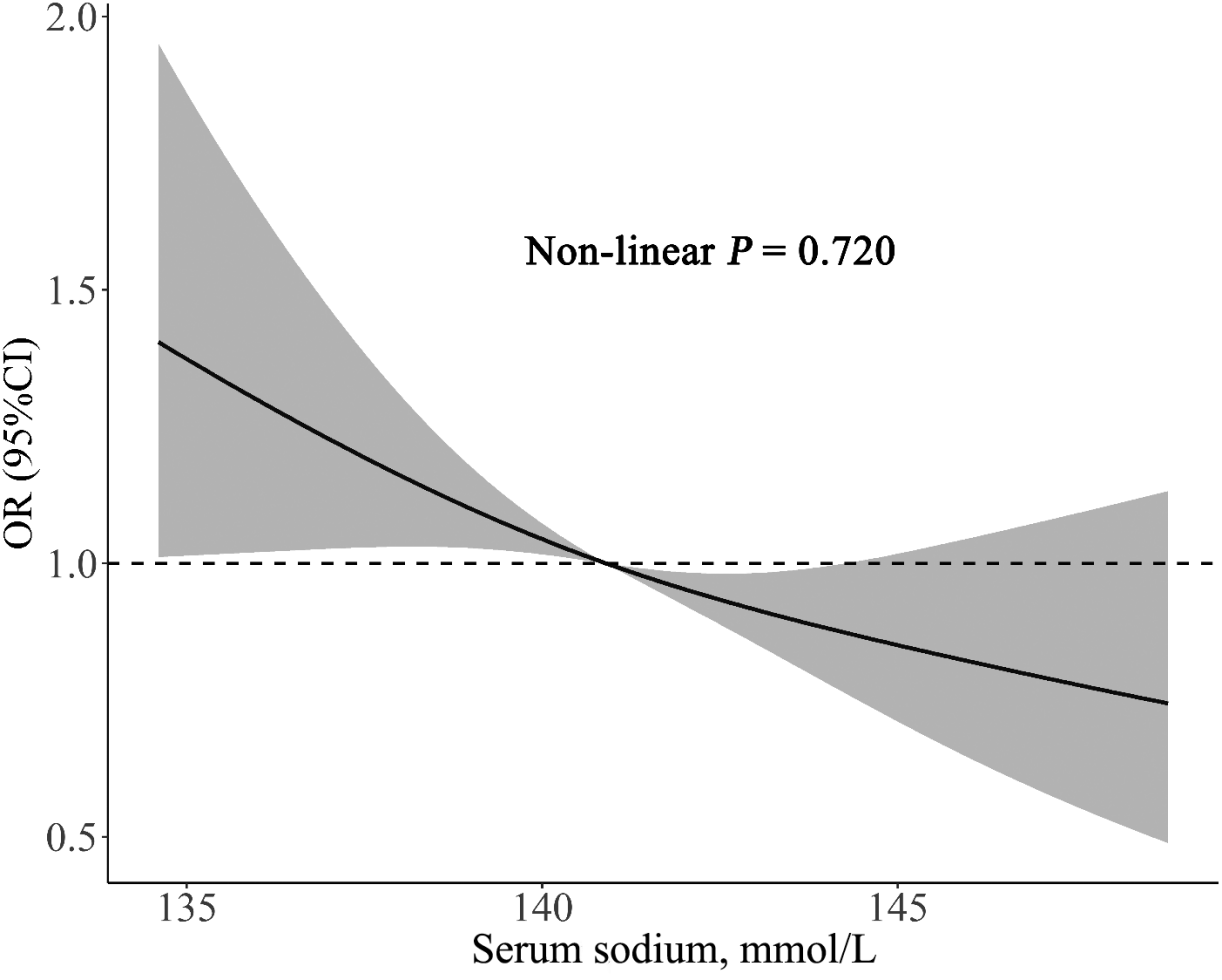
Restricted cubic spline analyses of new‐onset diabetes by serum sodium concentration. OR, odds ratio; CI, confidence interval. (Restricted cubic spline analyses were adjusted for age, gender, smoking, diastolic blood pressure, waist circumference, body mass index, fasting blood glucose, triglyceride, low‐density lipoprotein cholesterol, total cholesterol)

The logistic regression analysis results are presented in Table 3. When the serum sodium level was treated as a continuous variable, higher serum sodium was associated with a lower incidence of new‐onset diabetes. In model III, per 1‐SD increment of serum sodium was associated with 14% lower risk of new‐onset diabetes (odds ratio [OR] = 0.86; CI: 0.78, 0.97). The Q1 of serum sodium presented the highest risk of new‐onset diabetes. Compared to Q1, the OR for Q2, Q3, and Q4 were 0.84 (CI: 0.66, 1.08), 0.70 (CI: 0.54, 0.90), and 0.69 (CI: 0.53, 0.89), respectively. The restricted cubic spline (Figure 2) showed a linearly decreasing trend of diabetes risk with the elevation of serum sodium (nonlinear *p* = 0.720).

**TABLE 3:** Logistic regression analysis for the association of serum sodium with new‐onset diabetes

	Model I	*p* value	Model II	*p* value	Model III	*p* value
Serum sodium						
Per SD increment	0.85 (0.78, 0.92)	<0.001	0.86 (0.78, 0.94)	0.001	0.86 (0.78, 0.94)	0.001
Quartiles						
Q1	Ref		Ref		Ref	
Q2	0.88 (0.70, 1.10)	0.267	0.84 (0.66, 1.08)	0.172	0.84 (0.66, 1.08)	0.181
Q3	0.69 (0.54, 0.88)	0.003	0.70 (0.54, 0.90)	0.006	0.70 (0.54, 0.90)	0.006
Q4	0.68 (0.54, 0.87)	0.002	0.69 (0.53, 0.89)	0.005	0.69 (0.53, 0.89)	0.005
*p* for trend		<0.001		0.002		0.002

*Note*: Values are presented as odds ratios (95% confidence interval). Model I was adjusted for none; model II for age, gender, smoking, alcohol drinking, systolic blood pressure, diastolic blood pressure, waist circumference, body mass index, fasting blood glucose, triglycerides, low‐density lipoprotein cholesterol, high‐density lipoprotein cholesterol, total cholesterol, estimated glomerular filtration rate, serum potassium, serum calcium, antihypertensive medication; and model III for age, gender, smoking, diastolic blood pressure, waist circumference, body mass index, fasting blood glucose, triglycerides, low‐density lipoprotein cholesterol, total cholesterol.

Abbreviations: Q, quartile; SD, standard deviation.

### 3.3 Subgroup analysis

Table 4 presents the results of the subgroup analysis. Grouped by age, gender, BMI, FBG, eGFR, and hypertensive medication, the association of serum sodium with new‐onset diabetes was consistent across all the subgroups (*p* for interaction >0.05).

**TABLE 4:** Subgroup analysis

	*n*	OR (95% CI)	*p*	*p* for interaction
Age				0.706
Age < 60	2423	0.87 (0.77, 0.99)	0.031	
Age ≥ 60	2015	0.85 (0.74, 0.97)	0.015	
Gender				0.322
Female	2268	0.89 (0.78, 1.01)	0.072	
Male	2170	0.83 (0.72, 0.95)	0.007	
BMI				0.929
BMI < 24	1687	0.88 (0.74, 1.05)	0.151	
BMI ≥ 24	2751	0.84 (0.76, 0.94)	0.003	
FBG				0.576
FBG < 6.1	4189	0.87 (0.79, 0.96)	0.004	
FBG ≥ 6.1	249	0.85 (0.65, 1.12)	0.25	
eGFR				0.854
eGFR < 90	2274	0.87 (0.77, 0.99)	0.031	
eGFR ≥ 90	2164	0.84 (0.73, 0.96)	0.013	
Hypertensive medication				0.707
Yes	1265	0.86 (0.72, 1.03)	0.108	
No	3173	0.85 (0.76, 0.94)	0.002	

*Note*: When analyzing a subgroup variable, age, gender, smoking, diastolic blood pressure, waist circumference, body mass index, fasting blood glucose, triglycerides, low‐density lipoprotein cholesterol, and total cholesterol were adjusted, except the variable itself.

Abbreviations: BMI, body mass index; CI, confidence interval; eGFR, estimated glomerular filtration rate; FBG, fasting blood glucose; *n*, number; OR, odds ratio.

## 4 DISCUSSION

We found an inverse association of serum sodium with new‐onset diabetes in hypertensive patients in the present study. Per 3.39‐mmol/L increment of serum sodium was associated with 14% reduction of new‐onset diabetes.

There are plenty of studies investigating the impact of sodium intake on the occurrence and development of diabetes, but studies on serum sodium are lacking. Compared to sodium intake, serum sodium is a parameter that is easy to obtain, and it generally remains within a narrow range due to the highly regulated fluid homeostasis and genetic factors.^16^, ^20^ Therefore, it is of great clinical importance to study the relationship between serum sodium and diabetes. Consistent with our findings, Lago et al. found that serum sodium was inversely related to diabetes in a nonhypertensive population.^21^ Hou et al. reported that every 2‐mmol/L increment of serum sodium was associated with approximately 11% lower risk of incident diabetes in the general population aged ≥40 years.^22^ Our results showed that this inverse association also existed in the hypertensive population, but it was less significant than in the general population.

The possible mechanisms to explain this relationship were as follows. First, decreased serum sodium can activate the renin‐angiotensin‐aldosterone system (RAAS), resulting in increased renin, angiotensin, and aldosterone concentration. These components of RAAS can cause oxidative stress in pancreatic β‐cells, which decrease insulin secretion and exacerbate insulin resistance.^23^, ^24^ Serum sodium can be affected by dietary sodium intake.^10^ Transient or long‐term low‐sodium diets can increase RAAS components both in normotensive and hypertensive participants,^25^, ^26^, ^27^ and it is higher in normotensive than hypertensive participants.^28^ Considering that the RAAS is usually active in hypertensive patients, fighting against the deleterious axis of RAAS may be a feasible way to prevent hypertensive patients from developing diabetes.^29^ The RAAS blockers like angiotensin‐converting enzyme inhibitors and angiotensin II receptor blockers have been recognized as antihypertensive drugs that improve insulin sensitivity in hypertensive patients.^30^ Thus, the compensatory activation of the RAAS and induced insulin resistance by lower serum sodium should be considered, and this effect could be affected with RAAS inhibitor therapy in hypertensive patients.

Second, though moderate sodium reduction will not stimulate sympathetic outflow,^25^ a stricter sodium restriction can stimulate the secretion of norepinephrine and increase sympathetic nervous activity, thus worsening insulin sensitivity.^30^ The activation of the sympathetic nervous system causes skeletal muscle vasoconstriction, which reduces blood flow and insulin‐mediated glucose uptake, thus leading to insulin resistance.^31^

Third, serum sodium and serum glucose were two of the predominant substances that regulate the osmotic pressure of the human body. Hyponatremia is a common condition in diabetic patients, especially those with poor blood glucose control.^15^, ^32^, ^33^ Increased serum glucose may cause cells to move water outward and subsequently reduce serum sodium,^34^ and the interaction between insulin and vasopressin also contributes.^35^ Though the baseline levels of FBG in our study did not show significant differences among serum sodium quartiles, a small reduction in serum sodium possibly correlated with the impaired glucose tolerance that could have been overlooked. Further studies on the relationship between serum sodium and prediabetes are needed.

Fourth, we noticed that the groups with lower levels of serum sodium were older and had lower eGFR at baseline. Due to the decreased secretion of antidiuretic hormones and more comorbidities, hyponatremia is the most common electrolyte disorder in older people.^36^ The decline in eGFR is closely related to aging. Even in healthy persons, eGFR declines by about 0.75 ml/min per year.^37^ Contrary to our results, other studies have observed a positive cross‐sectional association between serum sodium and age, resulting from renal regulation changing with age.^21^, ^38^ In addition, some studies found that higher serum sodium is related to renal dysfunction and lower eGFR.^39^, ^40^ The different study populations may account for this discrepancy. Compared with these studies, our study population was consistently hypertensive and of relatively older age (mean age 58.65 ± 13.84 years). Therefore, the participants with lower serum sodium were older and more likely to develop other systemic diseases and diabetes. Obesity can cause both insulin resistance and pancreatic β‐cell dysfunction, resulting in the onset of diabetes.^41^ At baseline, the new‐onset diabetes group had an apparently higher BMI and waist circumference. But the obesity indexes did not differ significantly across the groups divided by serum sodium levels, and the results of the BMI subgroups were not significantly different. This might suggest that the association of serum sodium and new‐onset diabetes is independent of obesity.

Sodium is widely accepted as a risk factor that increases blood pressure.^11^, ^42^ However, in recent studies, serum sodium was also found to be inversely associated with cardiovascular risk and mortality.^12^, ^13^, ^14^ Sodium may have pleiotropic effects on the cardiovascular system and glucose metabolism.^43^ Higher serum sodium is associated with a higher risk of cardiovascular events in hypertensive patients, while lower serum sodium is associated with increased risk regardless of hypertension or nonhypertension.^44^ Moreover, lower serum sodium is significantly associated with a poor prognosis in diabetics.^45^ Despite the negative cardiovascular effects that higher serum sodium may cause in hypertensive individuals, we also found that hypertensive patients with relatively higher serum sodium had lower diabetes risk. This evidence suggests that sodium intake and serum sodium control may need to be further refined to provide specific cardiovascular and diabetes risk management for hypertensive patients.

Due to the fact that our study is a single‐center observational study, some inherent limitations exist. The variables included in this study were limited, and some information such as family history and duration of hypertension and diabetes was not collected completely. Consequently, there may be unadjusted confounding factors. Besides, the oral glucose tolerance test, glycosylated hemoglobin (HbA1c) test, and relative antibodies detection for type 1 diabetes were not performed. Therefore, some diabetes cases may be underdiagnosed, and type 1 and type 2 diabetes were not differentiated. Moreover, the applicability of our findings to hypertensive patients of other ethnic groups remains to be further explored, and future multicenter studies are expected.

## 5 CONCLUSION

In this study, an inverse association between serum sodium and new‐onset diabetes in hypertensive patients was observed. Further studies are needed to support our findings.

## DISCLOSURE STATEMENT

The authors declare no conflict of interest.

## Supporting information


**Table S1.** The classification of antihypertensive drugs and lipid‐lowering drugs at baseline
