# Supplementary material for: Serum sodium level is inversely associated with new‐onset diabetes in hypertensive patients
Source: J Diabetes. 2022 Dec 5;14(12):831–9. doi: 10.1111/1753-0407.13338 (PMC9789394; doi:10.1111/1753-0407.13338)
Supplement: Supplementary file 1 — Table S1. The classification of antihypertensive drugs and lipid‐lowering drugs at baseline [file JDB-14-831-s001.docx]

**Supplementary Table 1.** The classification of antihypertensive drugs and lipid-lowering drugs at baseline

|  | **Total** | **Non-diabetes group** | **New-onset diabetes group** | ***P*-value** |
| --- | --- | --- | --- | --- |
| n | 4438 | 3821 | 617 |  |
| Antihypertensive drugs |  |  |  |  |
| Diuretics | 353 (8.0) | 306 (8.0) | 47 (7.6) | 0.739 |
| Beta blockers | 454 (10.2) | 386 (10.1) | 68 (11.0) | 0.485 |
| CCBs | 1779 (40.1) | 1518 (39.7) | 261 (42.3) | 0.226 |
| ACEIs | 568 (12.8) | 479 (12.5) | 89 (14.4) | 0.193 |
| ARBs | 1854 (41.8) | 1602 (41.9) | 252 (40.8) | 0.613 |
| Lipid-lowering drugs |  |  |  |  |
| statins | 383 (8.6) | 340 (8.9) | 43 (7.0) | 0.113 |
| fibrates | 31 (0.7) | 26 (0.7) | 5 (0.8) | 0.719 |

Abbreviations: n, number; CCB, calcium channel blocker; ACEI, angiotensin-converting enzyme inhibitor; ARB, Angiotensin Receptor Blocker
